# Functional polymorphisms in LncRNA HOTAIR contribute to susceptibility of pancreatic cancer

**DOI:** 10.1186/s12935-019-0761-x

**Published:** 2019-02-28

**Authors:** Dawei Jiang, Liu Xu, Jianqi Ni, Jie Zhang, Min Cai, Lan Shen

**Affiliations:** Department of Hepatobiliary Surgery, The First Affiliated Hospital of Jiaxing University, No. 1882 Zhonghuan South Road, Jiaxing, 314001 Zhejiang People’s Republic of China

**Keywords:** Pancreatic cancer, Variation, HOTAIR

## Abstract

**Background:**

Pancreatic cancer (PC) remains one of the most aggressive cancers worldwide. However, genetic factors underlying PC susceptibility remain largely unclear. Long noncoding RNA (lncRNA) HOX transcript antisense RNA (HOTAIR) acts as an oncogene and its genetic variation has been linked to many cancers. However, the associations between genetic variants in HOTAIR gene and PC risk has not yet been reported.

**Methods:**

A two-stage, case–control study was conducted to investigate the associations between HOTAIR SNPs and the PC risk. Dual luciferase reporter assay and real-time -PCR (RT-PCR) was conducted to evaluate the potential regulatory function of HOTAIR rs4759314 and rs200349340.

**Results:**

We found the minor alleles of rs4759314 (OR = 1.76; 95 CI 1.37–2.25; P = 0.001) and rs200349340 (OR = 1.32; 95 CI 1.12–1.56; P = 0.001) were significantly associated with PC susceptibility. In functional experiments, we found subjects carrying the minor alleles of rs4759314 and rs200349340 had significantly higher HOTAIR RNA levels (mean ± SD) than those carrying the major alleles in PC tissues. For rs4759314, cells transfected with rs4759314 -G allele construct showed higher relative luciferase activity; while for rs200349340, cells transfected with rs200349340 -G allele construct showed more sensitive change of the relative luciferase activity.

**Conclusion:**

Our studies revealed that functional SNP rs4759314 and rs200349340 of HOTAIR had strong associations with PC susceptibility. These findings elucidate that functional genetic variants influencing lncRNA expression may explain a portion of PC genetic basis.

**Electronic supplementary material:**

The online version of this article (10.1186/s12935-019-0761-x) contains supplementary material, which is available to authorized users.

## Background

Pancreatic cancer (PC) remains one of the most aggressive cancers with a 5-year survival rate of less than 5%, and conventional treatment offers no hope of cure and has little palliative value [1, 2]. It was estimated that 55,440 new PC cases and 44,330 new PC deaths would occur in United States [3]. In contrast to the overall declining trends for the 4 major cancers (Lung, breast, prostate, and colorectum), death rates rose during 2011 through 2015 for PC in men by 0.3% per year [3]. In China, 90.1 thousands new PC cases and 79.4 thousands new PC deaths were estimated per year, according to the report of the National Office for Cancer Prevention and Control of China [4]. Although genome-wide association studies (GWASs) have identified several loci associated with PC risk; most of genetic factors remain unknown [5–7]. Therefore, identification and characterization of new genetic variations in predicting PC risk are essential.

Long non-coding RNAs (lncRNAs) are an important class of genes involved in various biological processes [8–10]. Among them, lncRNA HOX transcript antisense RNA (HOTAIR) was involved in pathogenesis and progress of multiple tumors, including PC [11–19]. Previous studies showed that HOTAIR could induce genome-wide retargeting of Polycomb repressive complex 2 (PRC2), contribute to altered H3 lysine 27 methylation, and further regulate various downstream genes and promote cancer cell metastasis [20, 21]. Significant associations between overexpression of HOTAIR and susceptibility, metastasis and/or poor prognosis of several human cancers such as liver, breast, pancreas, colon, lung, laryngeal, nasopharyngeal, and esophageal cancers have been reported [14, 22–24]. Meanwhile, studies showed that HOTAIR was significantly up-regulated in PC tissues, compared with normal pancreatic tissues [19, 25]. Furthermore, increasing publications are drawing attention to the associations between common polymorphisms in HOTAIR and risk of cancers, although meta-analysis showed these results had been controversial and inconsistent [26]. Even with the potential importance of HOTAIR in carcinogenesis, little has been investigated about the functional significance of HOTAIR locus with PC susceptibility.

Here, we hypothesized that genetic variants in the HOTAIR gene might affect HOTAIR expression and/or its function, which in turn, might influence consequential risk of developing PC. In current study, we conducted a two-stage, case–control study to investigate the associations between HOTAIR genotypes and PC risk.

## Patients and methods

### Study subjects

Totally 900 PC cases were collected and pathologically confirmed, while 900 matched controls were recruited in the same hospital. All the samples were continuously collected since Jan 2005. In stage 1, the samples were collected before Sep 2010. While the samples in stage 2 were collected from Oct 2010 to Jun 2016. The inclusion and exclusion criteria for PC patients were: pathologically confirmed and without treatment of any medications, while the inclusion and exclusion criteria for control group were: (1) without cancer or other serious illness; (2) matched with the PC patients by age (± 5 years), gender, and area of residence (urban and rural). Face-to-face interviews were conducted by trained interviewers using a structured questionnaire relating to demographic characteristics and lifestyle-related factors. Meanwhile, 10 mL peripheral blood from each individual was collected and stored in sodium citrate anticoagulant tubes at − 80 °C for DNA isolation. The study was performed in accordance with Declaration of Helsinki, and approved by the hospital’s Ethics Committee. All the participants provided signed informed consent before the study.

### SNP selection and genotyping

**Fig. 1:**
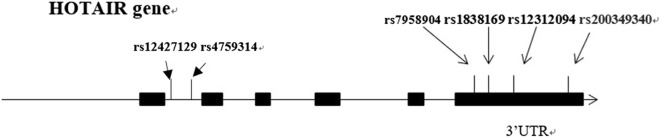
Relative location about the 6 candidate SNPs in HOTAIR gene

The tagSNPs covering the HOTAIR region were selected from 1000 genome CHB data (phase 3, minor allele frequency ≥ 5%, pairwise r2 ≥ 0.8) using the Haploview 4.2 software. We also considered the potential functional variants that were located in the binding site of miRNAs. Finally, six SNPs, including rs4759314, rs12427129, as well as four SNPs in 3′UTR of HOTAIR gene (rs200349340, rs12312094, rs1838169, and rs7958904), were finally selected as the candidates (Fig. 1). Genotyping was conducted using matrix assisted laser desorption ionization time-of-flight mass spectrometry (MALDI-TOF MS) (the mass spectrum plots for three genotypes of rs200349340 were presented in Additional file 1: Figures S1–S3). Genotype calling was conducted using MassARRAY RT software version 3.0. Genotyping data was then analyzed by MassARRAY Typer software 4.0.3 (Sequenom, San Diego, CA, USA) in conjunction with Sequenom’s iPLEX™ Gold assay. Quality control was detected using Sanger sequencing of ~ 10% randomly selected samples, yielding a 100% concordance.

### Real-time analyses of LncRNA HOTAIR

Total RNA was extracted from PC and adjacent normal tissues using TRIzol reagent (Invitrogen, Carlsbad, CA, USA). Then cDNA was synthesized with M-MLV reverse transcriptase (Invitrogen). Real-time reverse transcription-polymerase chain reaction (RT-PCR) with SYBR Green assay (TaKaRa Biotechnology, Dalian, China) was performed to examine expression level of HOTAIR (forward-primer: GGTAGAAAAAGCAACCACGAAGC; reverse-primer: ACATAAACCTCTGTCTGTGAGTGCC). Glyceraldehyde 3-phosphate dehydrogenase (GAPDH) was used as an internal control (forward-primer: CCGGGAAACTGTGGCGTGATGG; reverse-primer: AGGTGGAGGAGTGGGTGTCGCTGTT). The thermal cycling parameters were as follows: 95 °C for 5 min followed by 40 cycles of 95 °C for 30 s, 59 °C for 30 s, 72 °C for 30 s. The assay was conducted using the ABI 7300 system (Applied Biosystems). All reactions were done in triplicate and expression level of HOTAIR was calculated according to the equation 2^−∆∆Ct^.

### Dual luciferase reporter assay

**Fig. 2:**
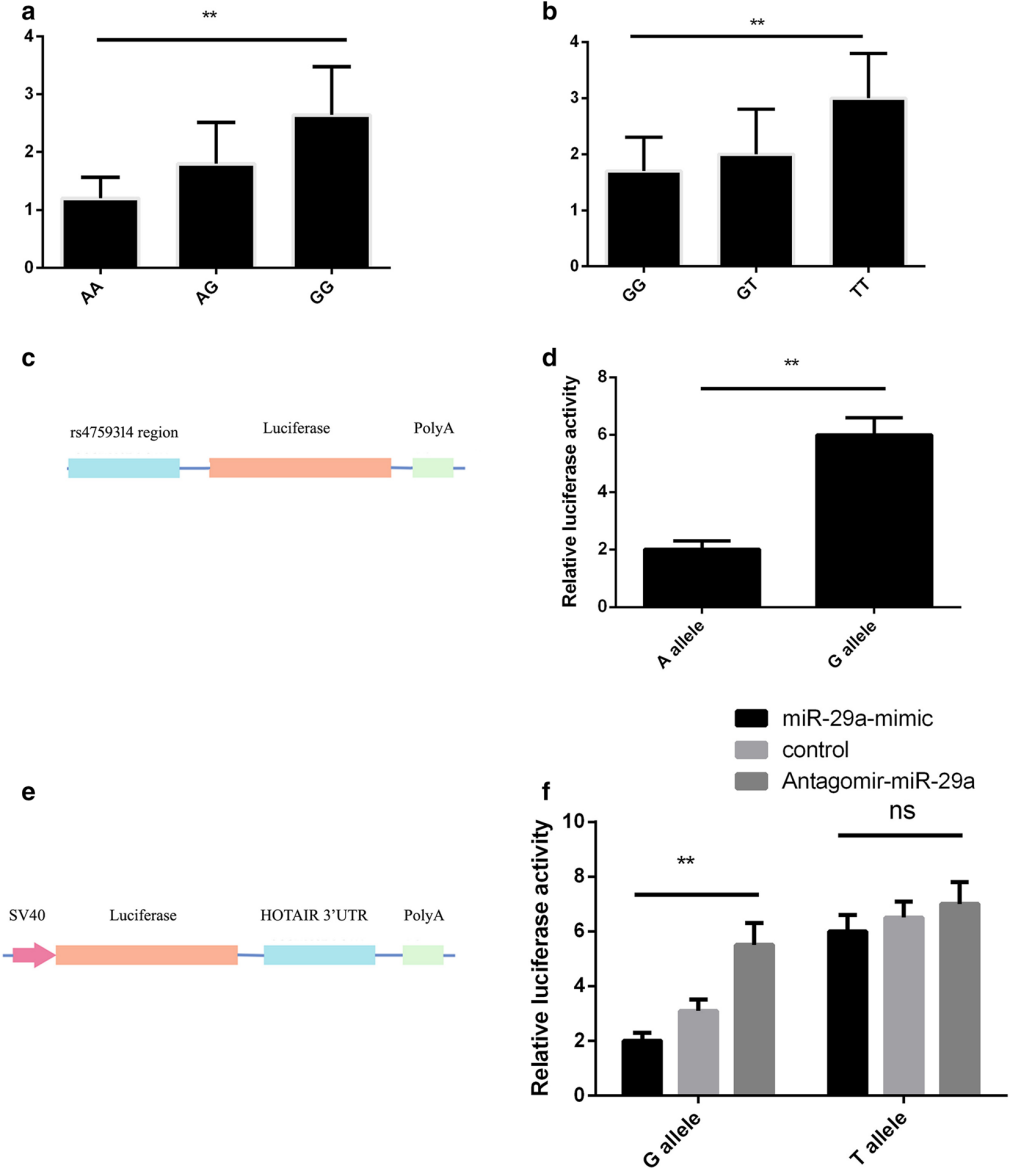
HOTAIR rs4759314 and rs200349340 affect luciferase activities in Sw1990 Cells. **a**, **b**: The expression of HOTAIR in carriers of different genotypes of rs4759314 and rs200349340 was calculated relative to expression of GAPDH using the 2^−∆∆Ct^ method.**c**: the schematic line diagram of the vector construct for rs4759314. **d**: relative luciferase activities (vs. Renila luciferase) of rs4759314 were measured in sw1990 cells transfected with A allele, or G allele. Three replicates for each group and the experiment were repeated three times; **e**: the schematic line diagram of the vector construct for rs200349340.**f**: relative luciferase activities (vs. Renila luciferase) of rs200349340 were measured in sw1990 cells transfected with G allele, or T allele. Cells in different groups were treated with control miR, miR-29a mimic or antagomir-miR-29a. Three replicates for each group and the experiment were repeated three times

To evaluate the potential function of HOTAIR rs4759314 and rs200349340, two reporter constructs carrying two copies of rs4759314 -A allele/G allele, rs200349340 -T allele/G allele were created based on pGL3-plasmids (Fig. 2c, e presents the schematic line diagrams). First, about 200-bp DNA sequences centered at rs4759314 (A or G allele) and rs200349340 (T or G allele) were synthesized; second, 2 tandem copies of these sequences were cloned into pGL-3 basic, pGL-3 promoter vectors (Promega, Madison, WI, USA), respectively. Third, cultured Sw1990 cells were transfected with 0.8 μg pGL-3 vectors (inserted with rs4759314 -A allele/G allele or rs200349340 -T allele/G allele), while tThe pRL-TK Renilla control vector (0.1 μg) (Promega, Madison, WI, USA) was also co-transfected as an internal control for transfection efficiency. Furthermore, for pGL-3 promoter vectors, Sw1990 Cells were transfected with mimic control, antagomir control, miR-29a mimic or antagomir miR-29a (GenePharma, Shanghai) in different groups. Finally, firefly and renilla luciferase activity was measured on Synergy H1 microplate reader (BioTek Instruments, Winooski, VT, USA) using Dual-luciferase Reporter Assay System (Promega, Madison, WI, USA) according to the instructions of manufactures. Luciferase activity was normalized to renilla activity to correct for differences in transfection efficiency. For each reporter construct, three independent transfection experiments were performed, and each was done in triplicate.

### Statistical analysis

Differences in the distribution of selected demographic variables and genotypes of SNPs were evaluated by Pearson’s χ^2^ test. Hardy–Weinberg equilibrium (HWE) for each SNP among controls was tested using a goodness-of-fit χ^2^-test. The associations of each SNP and PC susceptibility were estimated by using unconditional logistic regression analyses with odds ratios (ORs) and 95% confidence intervals (CIs). All analyses were conducted with SAS 9.3 software (SAS Institute, Inc., Cary, NC, USA), and a P value of less 0.05 for two-side was considered statistically significant.

## Results

### Characteristics of study subjects

In current study, a total of 900 PC cases and 900 controls were recruited in two stages, and there were no significant differences between PC cases and controls for each stage regarding to age, gender, drinking and smoking status (all P > 0.05, Table 1). However, we found PC cases are more likely to be diabetes patients in both stages (P < 0.001).

**Table 1:** Distribution of selected characteristics among PC cases and controls

Category	Stage 1			Stage 2		
	Cases (N = 400)	Controls (N = 400)	P value	Cases (N = 500)	Controls (N = 500)	P value
Age group						
< 50	200	192	0.572	245	239	0.704
≥ 50	200	208		255	261	
Gender						
Male	240	245	0.717	304	299	0.747
Female	160	155		196	201	
Drinking						
Never	271	279	0.542	333	350	0.248
Ever	129	121		167	150	
Smoking						
Never	291	300	0.469	351	369	0.205
Ever	109	100		149	131	
Diabetes						
Yes	102	50	*< 0.001*	137	66	*<* *0.001*
No	298	350		363	434	

Categorical variables: numbers, and P-values from x^2^ test

Italic values indicate significant of p value (P<0.05)

### Association of SNPs with PC susceptibility

Six SNPs, rs4759314, rs12427129, rs200349340, rs12312094, rs1838169, and rs7958904, were in accordance with Hardy–Weinberg equilibrium (HWE) in the control subjects. As shown in Table 2 and Table 3, we found that both SNP rs4759314 (OR = 1.84; 95 CI 1.26–2.67; P = 0.018) and rs200349340 (OR = 1.36; 95 CI 1.06–1.73; P = 0.014) were significantly associated with increased PC susceptibility, after adjusted for age, gender, Diabetes, drinking and smoking. Subsequently, we performed an independent validation set for the significant results and identified that rs4759314 and rs200349340 still had significant effects on PC risk (P < 0.05). When combined data of these two stages together, the minor alleles of rs4759314 (OR = 1.76; 95 CI 1.37–2.25; P = 0.001) and rs200349340 (OR = 1.32; 95 CI 1.12–1.56; P = 0.001) were significantly associated with PC susceptibility.

**Table 2:** Genetic variants of HOTAIR and PC risk in Stage 1

Genotype	Cases	Controls	Crude OR (95% CI)	Adjusted OR (95% CI)^a^
rs4759314				
AA	327	354	1.00 (reference)	1.00 (reference)
AG	65	44	1.60 (1.06–2.41)	1.66 (1.09–2.54)
GG	8	2	4.33 (1.04–18.1)	4.50 (1.11–18.3)
G vs A			1.76 (1.22–2.55)	1.84 (1.26–2.67)
P trend			*0.024*	*0.018*
rs12427129				
GG	359	356	1.00 (reference)	1.00 (reference)
AG	36	41	0.87 (0.54–1.39)	0.91 (0.66–1.25)
AA	5	3	1.65 (0.4–6.87)	1.72 (0.4–7.43)
A vs G			0.98 (0.64–1.49)	1.02 (0.76–1.35)
P trend			0.564	0.546
rs200349340				
GG	205	236	1.00 (reference)	1.00 (reference)
TG	161	140	1.32 (0.99–1.78)	1.38 (1.00–1.89)
TT	34	24	1.63 (0.94–2.83)	1.70 (0.96–2.99)
T vs G			1.31 (1.04–1.63)	1.36 (1.06–1.73)
P trend			0.061	*0.014*
rs12312094				
CC	260	278	1.00 (reference)	1.00 (reference)
CG	128	116	1.18 (0.87–1.6)	1.18 (0.87–1.60)
GG	12	6	2.14 (0.81–5.66)	2.13 (0.81–5.65)
G vs C			1.23 (0.95–1.59)	1.23 (0.89–1.70)
P trend			0.284	0.214
rs1838169				
CC	269	275	1.00 (reference)	1.00 (reference)
CG	122	118	1.06 (0.78–1.43)	1.10 (0.67–1.80)
GG	9	7	1.31 (0.48–3.57)	1.37 (0.46–4.06)
G vs C			1.07 (0.83–1.39)	1.12 (0.76–1.64)
P trend			0.721	0.577
rs7958904				
GG	215	237	1.00 (reference)	1.00 (reference)
CG	156	136	1.26 (0.94–1.7)	1.31 (0.94–1.82)
CC	29	27	1.18 (0.68–2.06)	1.23 (0.64–2.36)
C vs G			1.17 (0.94–1.47)	1.22 (0.93–1.59)
P trend			0.119	0.147

^a^Adjusting for age, gender, diabetes, drinking and smoking

Italic values indicate significant of p value (P<0.05)

**Table 3:** Associations of genetic variants of HOTAIR with PC risk in Stage 2 and combined results

Genotype	Cases	Controls	Crude OR (95% CI)	Adjusted OR (95% CI)^a^
rs4759314				
Stage 2				
AA	406	437	1.00 (reference)	1.00 (reference)
AG	83	59	1.51 (1.06–2.17)	1.57 (1.08–2.29)
GG	11	4	2.96 (0.99–8.89)	3.08 (1.04–9.13)
G vs A			1.63 (1.19–2.24)	1.7 (1.23–2.36)
P trend			*0.023*	*0.017*
Combined				
AA	733	791	1.00 (reference)	1.00 (reference)
AG	148	103	1.55 (1.18–2.03)	1.61 (1.22–2.13)
GG	19	6	3.42 (1.43–8.16)	3.55 (1.51–8.36)
G vs A			1.69 (1.33–2.15)	1.76 (1.37–2.25)
P trend			*0.001*	*0.001*
rs200349340				
Stage 2				
GG	264	295	1.00 (reference)	1.00 (reference)
TG	196	175	1.25 (0.96–1.63)	1.30 (0.97–1.74)
TT	40	30	1.49 (0.9–2.45)	1.54 (0.92–2.61)
T vs G			1.24 (1.01–1.52)	1.29 (1.03–1.62)
P trend			0.094	*0.027*
Combined				
GG	469	531	1.00 (reference)	1.00 (reference)
TG	357	315	1.28 (1.06–1.56)	1.33 (1.07–1.65)
TT	74	54	1.55 (1.07–2.25)	1.61 (1.10–2.37)
T vs G			1.27 (1.09–1.47)	1.32 (1.12–1.56)
P trend			*0.013*	*0.001*

^a^Adjusting for age, gender, diabetes, drinking and smoking

Italic values indicate significant of p value (P<0.05)

### Functional relevance of rs4759314 and rs200349340 on HOTAIR expression

We found that subjects with the minor alleles of rs4759314 and rs200349340 had significantly higher HOTAIR RNA levels (mean ± SD) than those with the major alleles in PC tissues (Fig. 2a, b, P < 0.01). Then dual luciferase reporter assay was conducted to evaluate the potential function of HOTAIR rs4759314 and rs200349340. For rs4759314, cells transfected with rs4759314 -G allele construct showed higher relative luciferase activity (Fig. 2c, d, P < 0.01); while for rs200349340, cells transfected with rs200349340 -G allele construct, miR-29a mimic showed more sensitive change of the relative luciferase activity (Fig. 2e, f, P < 0.01).

## Discussion

The current study explored the association between four functional SNPs located in 3′ UTR region of the HOTAIR gene and PC risk using a two-stage case–control study design. We found rs4759314 and rs200349340 were significantly associated with increased PC susceptibility. Functional studies revealed that minor alleles of rs4759314 and rs200349340 increased HOTAIR expression.

HOTAIR was one of the most studied lncRNAs in cancers [27–29]. LncRNA HOTAIR was first identified to impact H3K27me3 and breast cancer metastasis by Gupta et al. [30]. Since then, many studies have reported the function of HOTAIR regarding the pro-oncogenic and prognostic activities, radiosensitivity, biomarkers, cell proliferation, and metastasis of PC [18, 19, 25, 31–36]. Our study identified that expression of HOTAIR was significantly higher in PC tissues than that in adjacent normal tissues, which is consistent with the results of ONCOMINE (https://www.oncomine.org).

Genetic variants of lncRNA HOTAIR has been linked to many diseases, including cancers, congenital heart disease, lower extremity malformations, benign prostate hyperplasia, noise-induced hearing loss, lead poisoning, and so on [26, 37–47]. In 2014, Zhang et al. [37] first evaluated variants of HOTAIR in esophageal squamous cell carcinoma, and found SNP rs920778 that regulates the expression of lncRNA HOTAIR via a novel intronic enhancer. Following, it was linked to breast cancer, gastric cancer, colorectal cancer, lung cancer, cervical cancer, ovarian cancer, osteosarcoma, papillary thyroid carcinoma, hepatocellular carcinoma, glioma, prostate cancer, oral cancer, neuroblastoma [26, 42–44, 47, 48]. Li found rs4759314 polymorphism was associated with the risk of congenital heart disease by alternating downstream signaling via reducing its expression [38]. A significant association between polymorphism rs4759314 and susceptibility to gastric cancer in allele contrast was identified by an epidemiological investigation and a meta-analysis [21, 49]. This was similar to our results, that rs4759314 was significantly associated with increased PC susceptibility. Another interesting finding was that SNP rs200349340, resided in 3′UTR region of the HOTAIR gene, contributing to a genotype specific effect on the expression of lncRNA HOTAIR via interfering the binding of miR-29a. The miRNA-29 family, including miR-29a, miR-29b and miR-29c, was recently reported to be aberrantly expressed in multiple cancers [50]. Previously, miR-29a-3p were identified to be associated with prognosis of hepatocellular carcinoma [51]. Meanwhile, miR-29a was found to function as tumor suppressors by targeting the MUC1 mucin in pancreatic cancer cells [52]. To the best of our knowledge, the functional polymorphism- rs200349340 has not been reported before.

One of the major strengths of the current study is the large sample size, since with a total of 1800 subjects were included in this study. Our statistical power reached 99.5% for detecting an association for rs4759314, 95.5% for detecting an association for rs200349340. The second strength should be two-stage case–control study design, which makes the positive findings more reliable. Finally, the functional validation of the variant rs200349340 further convinced the results. Several limitations may also exist in the current study. For example, there might be inherent selection bias for the hospital-based study. Thus, the findings of our study are warranted to be validated in different populations. Also, we only obtained information on smoking, drinking and diabetes in the present study. Further studies should focus on more environmental risk factors, such as dietary habits, life style of the subjects, to make the result more informative, and to perform potential interaction analyses.

## Conclusions

In conclusion, we identified that functional SNP rs4759314 and rs200349340 of HOTAIR had strong associations with PC susceptibility. These indicated that lncRNA HOTAIR and its genetic variations may be a potential target for risk evaluation, early detection and therapeutic target of PC; and larger prospective and experimental studies were warranted to further validation.

## Additional file

**Additional file 1: Figure S1.** The mass spectrum plot for rs200349340 TT genotype. **Figure S2.** The mass spectrum plot for rs200349340 GT genotype. **Figure S3.** The mass spectrum plot for rs200349340 GG genotype.

## Abbreviations

PC: pancreatic cancer
lncRNA: long noncoding RNA
HOTAIR: HOX transcript antisense RNA
RT-PCR: real-time reverse transcription-polymerase chain reaction
PRC2: polycomb repressive complex 2
MALDI-TOF MS: matrix assisted laser desorption ionization time-of-flight mass spectrometry
