# Supplementary material for: Functional polymorphisms in LncRNA HOTAIR contribute to susceptibility of pancreatic cancer
Source: Cancer Cell Int. 2019 Feb 28;19:47. doi: 10.1186/s12935-019-0761-x (PMC6396528; doi:10.1186/s12935-019-0761-x)
Supplement: Supplementary file 1 — Additional file 1: Figure S1. The mass spectrum plot for rs200349340 TT genotype. Figure S2. The mass spectrum plot for rs200349340 GT genotype. Figure S3. The mass spectrum plot for rs200349340 GG genotype. [file 12935_2019_761_MOESM1_ESM.docx]

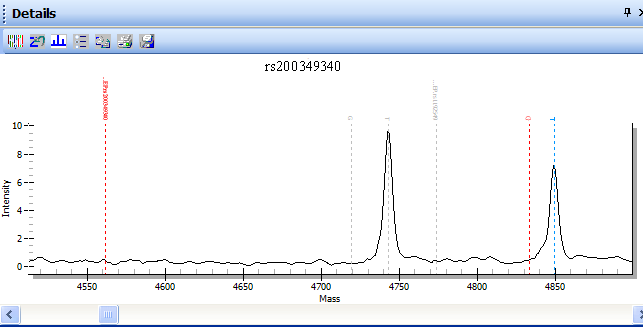


Figure S1 The mass spectrum plot for rs200349340 TT genotype


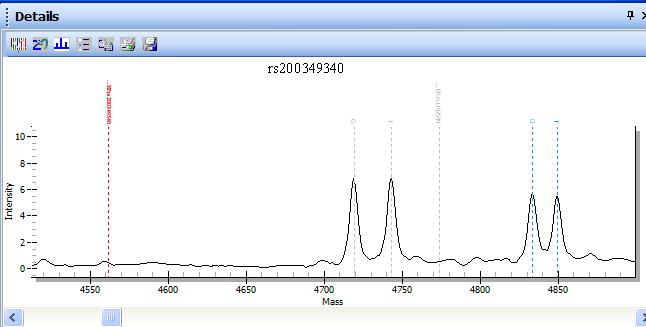


Figure S2 The mass spectrum plot for rs200349340 GT genotype


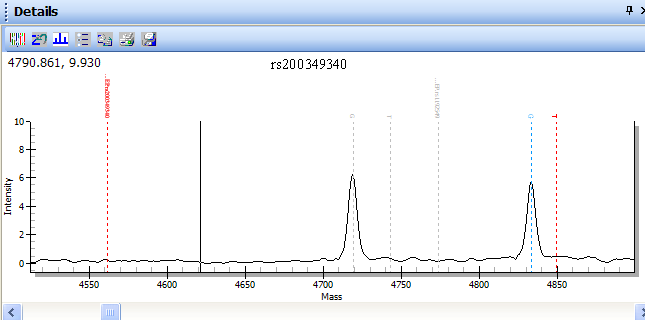


Figure S3 The mass spectrum plot for rs200349340 GG genotype
